# The effect of COVID-19 lockdown on lifestyle and mood in Croatian general population: a cross-sectional study

**DOI:** 10.3325/cmj.2020.61.309

**Published:** 2020-08

**Authors:** Zoran Đogaš, Linda Lušić Kalcina, Ivana Pavlinac Dodig, Sijana Demirović, Katarina Madirazza, Maja Valić, Renata Pecotić

**Affiliations:** 1 Department of Neuroscience, University of Split School of Medicine, Split, Croatia; 2 Split Sleep Medicine Center, Split, Croatia

## Abstract

**Aim:**

To investigate the effect of the coronavirus 2019 (COVID-19) lockdown on lifestyle behaviors and mood changes in the Croatian general population.

**Methods:**

During ten days of the COVID-19 lockdown in Croatia, 3027 respondents (70.3% female) from the general population completed an online, self-report questionnaire. Demographic data and data on lifestyle habits and mood changes before and during the COVID-19 lockdown were collected.

**Results:**

A total of 95.64% of respondents reported to follow most or all restrictions, with female sex (*P* < 0.001) and higher education level (*P* < 0.001) being associated with higher restriction compliance. Women smoked an increased number of cigarettes (*P* < 0.001). The proportion of respondents of both sexes who did not drink or drank 7 drinks per week or more increased (*P* < 0.001). Women also reported lower frequency (*P* = 0.001) and duration of physical exercise (*P* < 0.001). In total, 30.7% of respondents gained weight, with female sex (OR, 2.726) and higher BMI (OR, 1.116; both *P* < 0.001) being associated with an increased likelihood of gaining weight. Both men and women felt more frequently afraid (*P* < 0.001), discouraged (*P* < 0.001), and sad (*P* < 0.001).

**Conclusion:**

Public health authorities should promote the adoption of healthy lifestyles in order to reduce long-term negative effects of the lockdown.

The coronavirus disease-2019 (COVID-19) pandemic has tremendously changed our everyday life. Long-term home confinement and quarantine have affected daytime routines, working schedules (1), and lifestyle habits (2). The most likely scenario includes increases in poor-quality diet and sedentary time, with a decrease in physical activity (3,4). Sedentarism during the COVID-19 home confinement might detrimentally affect the neuromuscular system and, in combination with unhealthy diet (4,5), could lead to weight gain, blood pressure increase, and disturbed glucose tolerance, increasing the cardiovascular risk in the population (2,6,7). Although guidelines and interventions have been created to promote healthy lifestyles during the COVID-19 lockdown (2,5,6), it remains to be established whether and to what extent the countermeasures were adopted.

Unknown duration of the lockdown, fear of disease, boredom, lack of supplies, and misleading information might have all had a considerable psychological effect. Previous studies among quarantined people revealed increased emotional disturbances, anxiety, and general stress levels, and lower mood (8,9). Furthermore, the epidemic outbreak had detrimental effects on overall emotional well-being (10). However, the long-term effects of the COVID-19 lockdown on mood changes and psychological well-being of the general population are yet to be elucidated.

While the recent studies concentrated on the psychological impact of the quarantine (8), there is a need for more data about the general population’s self-care, nutritional status, physical activity, and sleep routines (4). Therefore, our study is the first to investigate lifestyle behaviors and mood changes in the Croatian general population during the COVID-19 lockdown.

## Material and methods

### Respondents

The study involved 3027 adult respondents (79.7% women) from the Croatian general population. The exclusion criterion was age less than 18. The distribution and sampling method prevented us from calculating the response rate.

### Methods and instrument

During ten days of the COVID-19 lockdown in Croatia, from April 25 to May 5, 2020, an online, self-report questionnaire (Supplementary material)(web extra material 1) was distributed through social media platforms and email messages to authors’ contacts (snowball sampling technique). All contacts were asked to forward the link to the survey to their contacts. Data collection was ended on May 5, when quarantine measures in Croatia ended. The study protocol was approved by the Biomedical Research Ethics Committee at the University of Split School of Medicine (2181-198-03-04-20-0056).

### Statistical analysis

The normality of distribution was tested with the Kolmogorov-Smirnov test. Data are presented as mean ± standard deviation or median and interquartile range. All *P* values were adjusted for multiplicity using Bonferroni adjustment. The U statistic of the Mann-Whitney test and χ^2^ statistic for the Kruskal-Wallis test were reported. Ordinal variables were dummy coded and contrasted to the last category in the analysis. Odds ratio and 95% confidence intervals were calculated in the logistic regression. Statistical significance was set at *P* < 0.05. Data analysis was performed with SPSS, version 14 (IBM, Armonk, NY, USA).

## Results

### Demographic data and restrictions compliance

Demographic characteristics are summarized in Table 1. During the COVID-19 lockdown, the majority of the participants experienced changes in work rhythm, such as working from home (40.78%), rotating shift work (15.28%), part-time working hours (5.13%), or other (Table 1).

**Table 1:** Demographic data of study respondents*

	Total N = 3027	Men N = 506	Women N = 1989
**Age (median, IQR)**	40 (30-50)	42 (31-52)	39 (30-49)
**Body weight (mean ± SD)**	74.03 ± 16.03	90.74 ± 15.72	69.05 ± 12.52
**Height (mean ± SD)**	172.84 ± 8.66	183.38 ± 7.61	169.76 ± 6.40
Body mass index **(mean ± SD)**	24.64 ± 4.22	26.90 ± 4.25	23.94 ± 4.00
**Education (no, %)**			
elementary school	20 (0.80)	2 (0.40)	18 (0.90)
high school	687 (27.54)	129 (25.49)	558 (28.05)
college or bachelor degree	412 (16.51)	81 (16.01)	331 (16.64)
master's degree	1137 (45.57)	210 (41.50)	927 (46.61)
PhD	239 (9.58)	84 (16.60)	155 (7.79)
**Working status (no, %)**			
employed	2208 (73.09)	391 (77.27)	1428 (71.83)
unemployed	535 (17.71)	79 (15.61)	373 (18.76)
retired	152 (5.03)	34 (6.72)	79 (3.97)
maternity leave	126 (4.17)	2 (0.40)	108 (5.43)
**Work rhythm during lockdown (no, %)**			
no change in work rhythm	221 (12.06)	95 (20.83)	180 (10.58)
work from home	747 (40.78)	176 (38.60)	702 (41.27)
rotating shift work	280 (15.28)	68 (14.91)	264 (15.52)
part-time working hours	94 (5.13)	16 (3.51)	100 (5.88)
other	490 (26.75)	101 (22.15)	455 (26.75)
**Living conditions during lockdown (no, %)**			
alone	295 (9.75)	65 (12.85)	169 (8.50)
with a partner	510 (16.85)	79 (15.61)	337 (16.95)
with partner and children	1038 (34.29)	203 (40.12)	675 (33.95)
with children	178 (5.88)	6 (1.19)	123 (6.19)
with partner, children, and elderly	450 (14.87)	73 (14.43)	302 (15.19)
other	556 (18.37)	80 (15.81)	382 (19.22)

*IQR – interquartile range; SD – standard deviation.

A total of 95.64% of respondents reported to follow most of or all the restrictions. Women followed all the restrictions more frequently than men (59.1% vs 49.4%; U = 453170, *P* < 0.001). Restriction compliance depended on the education level (χ^2^ (2) = 20.304; *P* < 0.001). The respondents following all the restrictions had a higher education level than respondents following most of the restrictions (U = 623523; *P* < 0.001), some of the restrictions (U = 57348.5; *P* = 0.024), and no restrictions (U = 5990.5; *P* = 0.036). Respondents with a master’s degree and a PhD (60.2% and 64.4%) obeyed all restrictions more frequently than respondents with a college degree (54.6%), high school degree (51.1%), and elementary school degree (55%). Respondents with different levels of restrictions compliance did not significantly differ in working status and average age (χ^2^ (3) = 5.937; *P* = 0.115, χ^2^ (3) = 4.465; *P* = 0.215, respectively).

### Lifestyle habits

Women reported smoking a significantly increased number of cigarettes (from 11.8 ± 7.4 cigarettes/day before the lockdown to 13.9 ± 9.8 cigarettes/day during the lockdown, *P* < 0.001, Table 2). Men drank fewer cups of coffee (2.0 ± 1.2 cups/day vs 2.4 ± 1.2 cups/day, respectively, *P* < 0.001, Table 2). The proportion of respondents of both sexes who did not drink alcohol or drank up to 7 drinks per week or more increased, while the proportion of respondents who drank occasionally (once per month or up to 3 drinks per week) decreased (*P* < 0.001, Table 2). Similarly, the proportion of men who did not drink and those who drank more than 15 drinks per week increased, while the proportion of men drinking up to 15 drinks per week decreased (*P* < 0.001, Table 2). The proportion of women who did not drink and those who drank more than 3 drinks per week increased, while the proportion of women who drank once monthly and up to 3 drinks per week decreased (*P* < 0.001, Table 2).

**Table 2:** Lifestyle habits before and during the coronavirus disease-2019 lockdown*

	Before lockdown	During lockdown	P
**All participants**			
**Cigarettes daily count**	12.3 ± 7.8	14.3 ± 10.3	<0.001^†^
**Coffee cups daily count**	2.1 ± 1.0	2.1 ± 1.1	0.003^†^
**Frequency of exercise weekly**	2.8 ± 1.1	2.6 ± 1.2	<0.001^†^
**Duration of exercise (min)**	57.9 ± 34.5	51.1 ± 37.7	<0.001^†^
**Alcohol (no, %)**			
never	531 (19.1)	910 (32.1)	<0.001^‡^
once monthly	887 (31.9)	633 (22.3)	
up to 3 drinks weekly	898 (32.3)	773 (27.2)	
up to 7 drinks weekly	358 (12.9)	378 (13.3)	
up to 15 drinks weekly	76 (2.7)	96 (3.4)	
more than 15 drinks weekly	30 (1.1)	49 (1.7)	
**Men**			
**Cigarettes daily count**	14.1 ± 9.0	14.5 ± 11.5	>0.999^†^
**Coffee cups daily count**	2.4 ± 1.2	2.0 ± 1.2	<0.001^†^
**Frequency of exercise weekly**	2.8 ± 1.1	2.7 ± 1.2	0.453^†^
**Duration of exercise (min)**	61.2 ± 40.1	59.2 ± 55.9	>0.999^†^
**Alcohol (no, %)**			
Never	40 (8.4)	99 (20.5)	<0.001^‡^
once monthly	88 (18.4)	63 (13)	
up to 3 drinks weekly	178 (37.2)	144 (29.8)	
up to 7 drinks weekly	117 (24.4)	117 (24.2)	
up to 15 drinks weekly	39 (8.1)	33 (6.8)	
more than 15 drinks weekly	17 (3.5)	27 (5.6)	
**Women**			
**Cigarettes daily count**	11.8 ± 7.4	13.9 ± 9.8	<0.001^†^
**Coffee cups daily count**	2.1 ± 1.0	2.1 ± 1.1	>0.999^†^
**Frequency of exercise weekly**	2.8 ± 1.0	2.7 ± 1.2	0.001^†^
**Duration of exercise (min)**	55.6 ± 29.8	49.2 ± 32.5	<0.001^†^
**Alcohol (no, %)**			
never	387 (21.5)	653 (35.2)	<0.001^‡^
once monthly	658 (36.5)	474 (25.5)	
up to 3 drinks weekly	559 (31)	486 (26.2)	
up to 7 drinks weekly	167 (9.3)	187 (10.1)	
up to 15 drinks weekly	23 (1.3)	43 (2.3)	
more than 15 drinks weekly	8 (0.4)	14 (0.8)	

*Data are presented as mean ± standard deviation for continuous variables, or frequencies (percentages).

†t-test for paired samples.

‡Wilcoxon signed rank test.

During the pre-lockdown period, a higher proportion of men exercised compared with women (71.6% vs 60.3%, *P* < 0.001). Only in women, the frequency (from 2.8 ± 1.0 times per week before the lockdown to 2.7 ± 1.2 times per week during the lockdown, *P* = 0.001) and duration of physical exercise decreased (from 55.6 ± 29.8 min prior to lockdown to 49.2 ± 32.5 min during the lockdown, *P* < 0.001, Table 2).

### Body weight changes during COVID-19 lockdown

A total of 939 (30.7%) respondents reported having gained weight. Female sex (OR = 2.726) and higher BMI (OR = 1.116; both *P* < 0.001) were associated with an increased likelihood of gaining weight (Table 3). Exercising before the lockdown (OR = 0.756; *P* = 0.004) decreased the likelihood of gaining weight. The frequency of drinking alcohol (*P* = 0.154) and education level (*P* = 0.116) did not affect the odds of gaining weight (Table 3).

**Table 3:** Factors contributing to weight gain during the coronavirus disease-2019 (COVID-19) lockdown*

	B	P	Odds ratio (OR)	95% confidence interval for OR	
				lower	upper
**Body mass index**	0.110	<0.001	1.116	1.086	1.147
**Age**	-0.006	0.119	0.994	0.986	1.002
**Sex**	1.003	<0.001	2.726	2.043	3.636
**Education level**		0.116			
**elementary school**	0.611	0.262	1.842	0.634	5.356
**high school**	0.362	0.061	1.436	0.984	2.095
**college or bachelor's degree**	0.450	0.029	1.568	1.048	2.345
**master's degree**	0.473	0.009	1.606	1.127	2.287
**Exercise before COVID-19**	-0.280	0.004	0.756	0.625	0.915
**Frequency of drinking alcohol**		0.154			
**never**	0.454	0.362	1.575	0.593	4.180
**once monthly**	0.171	0.729	1.187	0.450	3.128
**up to 3 drinks weekly**	0.407	0.408	1.503	0.573	3.946
**up to 7 drinks weekly**	0.357	0.477	1.429	0.535	3.817
**up to 15 drinks weekly**	0.721	0.196	2.057	0.689	6.139

*Nagelkerke R^2^ **=** 7.2%, logistic regression model significant at *P* < 0.001.

### Mood changes

**Figure 1:**
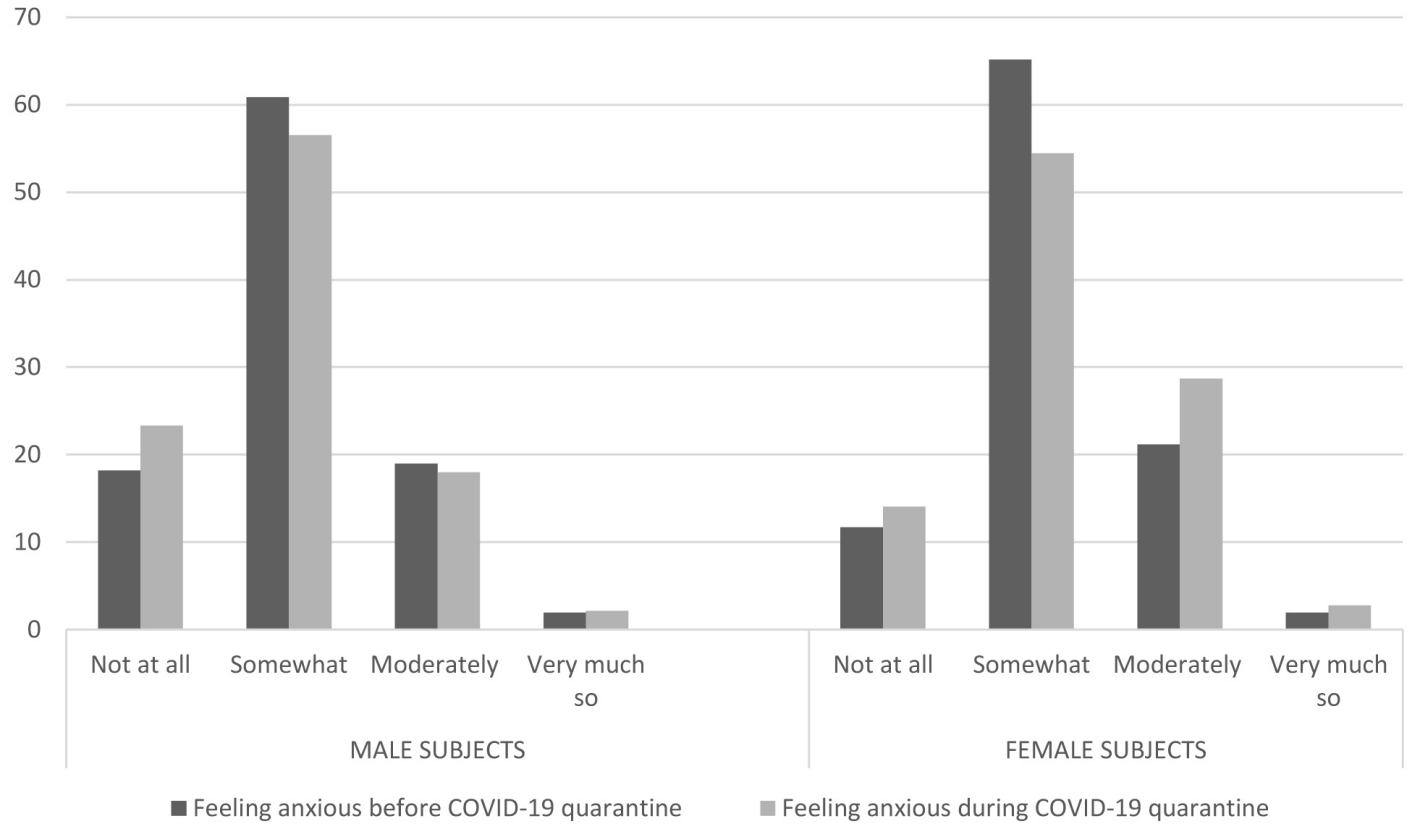
Difference in the frequency of anxiety before and during coronavirus disease-2019 (COVID-19) in men and women.

The respondents felt calm (*P* = 0.012) and content (*P* < 0.001, Table 4) more often before the lockdown than during the lockdown. However, during the lockdown, they more frequently felt rested (*P* < 0.001), but also more frequently afraid (*P* < 0.001), discouraged (*P* < 0.001), and sad (*P* < 0.001, Table 4). A higher proportion of women felt moderately and very anxious (*P* = 0.004), while no significant differences in anxiety were observed in men (Table 4 and Figure 1). There was an increase in the frequency of anxiety (*P* = 0.001) and a decrease in the frequency of calmness (*P* < 0.001) only among the respondents from Zagreb, who also experienced an earthquake during the lockdown period, whereas other respondents reported no significant change (Table 5).

**Table 4:** Mood changes before and during coronavirus disease-2019 (COVID-19) lockdown*

	Before COVID-19 lockdown	During COVID-19 lockdown	P^†^		
			all participants	women	men
**How often do you feel calm?**	3 (2-3)	3 (2-3)	0.012	0.077	>0.999
**How often do you feel rested?**	2 (2-3)	3 (2-3)	<0.001	<0.001	<0.001
**How often do you feel content?**	3 (2-3)	2 (2-3)	<0.001	<0.001	<0.001
**How often do you feel anxious?**	2 (2-2)	2 (2-3)	0.041	0.004	>0.999
**How often do you feel angry?**	2 (2-2)	2 (1-2)	>0.999	>0.999	>0.999
**How often do you feel afraid?**	1 (1-2)	2 (1-2)	<0.001	<0.001	<0.001
**How often do you feel discouraged?**	2 (1-2)	2 (1-3)	<0.001	<0.001	<0.001
**How often do you feel sad?**	2 (1-2)	2 (1-2)	<0.001	<0.001	<0.001

*All questions were answered on a Likert scale 1-4.

†two-related sample Wilcoxon signed rank test.

**Table 5:** Mood changes before and during coronavirus disease-2019 (COVID-19) among respondents experiencing Zagreb earthquake compared to others*

	Respondents with no earthquake experience during lockdown (N = 2536, 83.8%)			Respondents in Zagreb with earthquake experience during lockdown (N = 491, 16.2%)		
	before COVID-19 lockdown	during COVID-19 lockdown	P^†^	before COVID-19 lockdown	during COVID-19 lockdown	P^†^
**How often do you feel calm?**	3 (2-3)	3 (2-3)	0.814	3 (2-3)	2 (2-3)	<0.001
**How often do you feel rested?**	2 (2-3)	3 (2-3)	<0.001	2 (2-3)	3 (2-3)	<0.001
**How often do you feel content?**	3 (2-3)	2 (2-3)	<0.001	3 (2-3)	2 (2-3)	<0.001
**How often do you feel anxious?**	2 (2-2)	2 (2-3)	>0.999	2 (2-2)	2 (2-3)	0.001
**How often do you feel angry?**	2 (2-2)	2 (1-2)	>0.999	2 (2-2)	2 (1-2)	>0.999
**How often do you feel afraid?**	1 (1-2)	2 (1-2)	<0.001	1 (1-2)	2 (1-3)	<0.001
**How often do you feel discouraged?**	2 (1-2)	2 (1-3)	<0.001	1.5 (1-2)	2 (1-3)	<0.001
**How often do you feel sad?**	2 (1-2)	2 (1-2)	<0.001	2 (1-2)	2 (1-2)	<0.001

*All questions were answered on a Likert scale 1-4.

†Two-related sample Wilcoxon signed rank test.

## Discussion

In our study, respondents from the Croatian general population experienced lifestyle habits and mood changes during the COVID-19 lockdown. More precisely, women smoked more cigarettes per day, and the proportion of occasional alcohol drinkers of both sexes decreased and the proportion of those who do not drink or drink up to 7 drinks per week increased. The factors that increased the likelihood of gaining body weight were female sex, age from 30 to 49 years, college or master’s degree, and more frequent alcohol consumption. Furthermore, the lockdown was associated with mood changes in terms of more pronounced restlessness, fear, discouragement, and sadness.

Our respondents followed most or all of the pandemic restrictions implemented by the government, which indicates a general trust in Croatian health authorities' COVID-19 outbreak management. However, the compliance with restrictions such as social distancing and home confinement can negatively affect work and home schedules (1), mental health (8), healthy lifestyle, and nutritional habits (2,11). Since women and respondents with a higher education level had a better restriction compliance, their lifestyle habits might have been more affected.

Women reported a decreased frequency and duration of physical activity. Similarly, fewer women than men exercised even before the lockdown, which is in accordance with previous findings (12). The COVID-19 lockdown might accentuate the pre-existing sedentarism pandemic, and sedentary behavior might persist after the lockdown (13,14). Prolonged home confinement may lead to excessive physical inactivity (11,15), characterized as a major risk factor for cardiovascular and all-cause mortality (16-21) and associated with mood and mental health deterioration (22). Despite the World Health Organization’s clear recommendations and guidelines on maintaining at least minimum physical activity during this critical period (23), and high accessibility of tools and guidance videos (24), engaging in regular exercise remains challenging (13). Therefore, health professionals should emphasize the beneficial roles of daily exercise, such as antioxidant and anti-inflammatory processes promotion (25), stress alleviation (22,26), and immune system defense activity enhancement (27). Thus, considering the negative consequences of reduced physical activity and sedentary behavior during the COVID-19 quarantine (15,28), it is of major relevance to assess lifestyle changes during home confinement, especially in women. Effective interventions might help this vulnerable group maintain healthy body weight and reduce the risk factors for COVID-19.

In our study, 30.7% of respondents reported to have gained weight during the lockdown. This is not surprising considering the limited availability of fresh food and increased consumption of long-lasting and packaged food (2). The Mediterranean diet, rich in antioxidants, consumed in some parts of Croatia has highly protective cardiovascular effects (29,30). Still, during the lockdown a switch from the Mediterranean to an unhealthy diet might have occurred (2,31), possibly leading to increased oxidative stress and weight gain observed in our study (32-34).

Stress-related eating and drinking behavior has been identified as a way of stress management, especially in situations when there is a lack of social and emotional support (35-37). These oral behaviors are gender-specific, with women more frequently indulging in excessive eating and men in excessive drinking or smoking (35). In our study, a significant rise in anxiety levels in women might have led to an higher intake of unhealthy food. Indeed, women in our study showed an increased likelihood for gaining weight. It has been previously reported that women are more prone to adverse effects of sedentary behavior and unhealthy diet due to detrimental effects of estrogen level alterations on body weight, muscle mass, insulin resistance, oxidative stress, and blood pressure (12). Furthermore, taking into account the paradoxically low female adherence to healthy lifestyles (12), interventions addressing specifically women should be implemented.

During the lockdown our respondents smoked an increased number of cigarettes. However, little is known about smoking habits during the COVID-19 pandemic. During the first months, the number of searches for smoking cessation help on Google did not increase (38). It has been previously shown that quarantine may lead to smoking relapse (8), as well as increased food intake and obesity (39). Obesity and smoking can both worsen the symptoms and complications in COVID-19 patients and make individuals more vulnerable to the disease (40-43). Health professionals should develop adequate measures to avoid further increase in smoking rates and mitigate the consequences of the lockdown. Moreover, decreasing the detrimental effects of smoking and weight gain during the COVID-19 pandemic may foster the long-term sustainability of health care systems.

Stress has been reported to increase the desire for alcohol because of the dysregulation of the hypothalamic-pituitary-adrenal axis and neuroadaptations in stress and reward pathways caused by chronic alcohol consumption (44). Increased neuroendocrine and behavioral reactivity as a consequence of social isolation was shown even in animal experimental models (45), but the relationship between stress and social isolation in the general population during COVID-19 is unique and remains unclear, especially when considering alcohol use (46). Moreover, a complex association of excessive alcohol consumption and immune pathways impairments has been long observed, along with alcohol's immunosuppressive effects, such as increased susceptibility to pneumonia (47). Even though our results showed a decreased proportion of occasional drinkers, there was an increased proportion of those who drink up to than 7 drinks per week or more. This result emphasizes the need for issuing public health warnings about alcohol abuse.

Earthquake survivors are reported to develop PTSD, anxiety, and depression symptoms (48). This might explain the increase in the frequency of anxiety among respondents from Zagreb, who experienced an earthquake during the COVID-19 pandemic.

Several potential limitations of this study have to be mentioned. The majority of the respondents were highly educated women with internet access, which may limit the applicability of the results to the general population. Indeed, women were previously reported to show a decreased adherence to healthy lifestyle habits (12). However, as during the lockdown men and women could have more equally divided household and family responsibilities due to the decreased occupational workload, gender-related differences in pre-lockdown lifestyle habits were not the only factors that influenced lifestyle changes (12). Women were reported to be especially vulnerable to more severe psychological distress during quarantine (49), probably due to the previously reported greater health awareness (50). The higher level of perceived stress in women might add to their increased anxiety levels in this study. Therefore, we believe that a more equal gender distribution in our study would not have substantially changed the findings. In fact, the unequal gender distribution might be considered an important finding, as it indicates that women were more willing to participate in the study. Other potential sources of bias are self-assessment and snowball sampling technique. However, the chain referral process allowed for a rapid questionnaire distribution and capturing the lockdown-related changes at their peak. In addition, a large number of participants was accessed due to the fast and timely response. Furthermore, social distancing at the time of data collection prevented us from conducting studies involving a direct contact with respondents.

Although the population targeted with our sampling technique may not serve as a representative sample of the whole population, it may represent a growing segment of the population. More detailed subgroup analyses can contribute to designing a tailored approach in the current and future infectious outbreaks. Furthermore, we did not collect and analyze data on nutritional habits, which could elucidate the intertwined relationship of food intake, physical exercise, and body weight changes. Finally, we gathered no data on COVID-19 positivity.

In conclusion, our study was the first to provide data on lifestyle behaviors during the COVID-19 quarantine in Croatia, showing a decrease in physical activity and increase in smoking and body weight in a sample from the Croatian general population. These findings indicate that low dietary quality, lack of physical exercise, and increased tobacco and alcohol consumption might deteriorate overall and mental health outcomes and increase the global disease burden. Despite the Croatian general population’s great compliance with pandemic restrictions, our findings warrant the introduction of simultaneous lifestyle interventions. Thus, public health authorities should promote the adoption of healthy lifestyles in order to reduce long-term negative effects of the lockdown.
