# Supplementary material for: The effect of COVID-19 lockdown on lifestyle and mood in Croatian general population: a cross-sectional study
Source: Croat Med J. 2020 Aug;61(4):309–18. doi: 10.3325/cmj.2020.61.309 (PMC7480750; doi:10.3325/cmj.2020.61.309)
Supplement: Supplementary Table 1 [file CroatMedJ_61_s001.pdf]

Supplementary Material

The study questionnaire about lifestyle habits and mood changes before and during the COVID-19 quarantine including Demographics, Lifestyle habits and Mood self-assessment section

THE DEMOGRAPHICS SECTION							
**Year of birth**							
**Gender**	Male	Female	Other				
**Please, type the name of the city you currently (during the pandemic) live in.**							
**Please, indicate the highest level of your current education.**	Elementary school	High school	College	Bachelor degree	Master degree	PhD	
**Please, indicate your current employment status.**	Employed (full or part time)	Unemployed	Retired	Sick leave	Maternity leave		
**If you are currently employed, how are you working?**	I am working in similar conditions as before	I am working from home	I am working in rotating shifts due to pandemic	I work part time due to pandemic	Other		
**For how long have you been working in this manner (days)?**							
**Who do you live with during the COVID-19 pandemic?**	Alone	With partner and children	With children	With a partner	With a roommate	With family including partner, children and grandparents	Other
**Please, indicate how strictly are you following the restrictions due to COVID-19 pandemic?**	Not at all	Occasionally I do follow them	Most of the time I do follow them	I am following all the restrictions			
**Please, indicate your body mass in kilograms.**							
**During the quarantine, you have:**	lost weight	gained weight	remained the same weight				
**Please, indicate your height in centimeters.**							

THE LIFESTYLE HABITS SECTION							
**Do you smoke cigarettes?**	Yes			No			
**If yes, for how long have you been smoking cigarettes?**	Less than a year	1 to 5 years	5 to 10 years	10 to 20 years	Over 20 years		
**If yes, have you been smoking more cigarettes DURING the quarantine?**	Yes	No					
**How many cigarettes have you been smoking per day BEFORE the quarantine?**							
**How many cigarettes are you smoking per day DURING the quarantine?**							
**Do you drink coffee?**	Yes			No			
**If yes, how many cups of coffee have you been drinking per day BEFORE the quarantine?**							
**If yes, how many cups of coffee have you been drinking per day DURING the quarantine?**							
**How many alcoholic drinks have you been drinking BEFORE the quarantine?**	None	One in a month	Up to 3 drinks per week	Up to 7 drinks per week	Up to 15 drinks per week	More than 15 drinks per week	Other
**How many alcoholic drinks have you been drinking DURING the quarantine?**	None	One in a month	Up to 3 drinks per week	Up to 7 drinks per week	Up to 15 drinks per week	More than 15 drinks per week	Other
**Have you been exercising BEFORE the quarantine?**	Yes	No					
**If yes, how often have you been exercising BEFORE the quarantine?**	Daily	1-2 times per month	1-2 times per week	2-3 times per week	4-5 times per week		
**If yes, for how many minutes per day did you exercise?**							
**Have you been exercising DURING the quarantine?**	Yes	No					
**If yes, how often do you exercise DURING the quarantine?**	Daily	1-2 times per month	1-2 times per week	2-3 times per week	4-5 times per week		
**If yes, for how many minutes per day do you exercise?**							
**BEFORE the quarantine, how many hours per day did you spend:**							
**Watching TV?**	Less than 30 minutes	30 minutes to 1 hour	1 to 2 hours	2 to 3 hours	3 to 4 hours	More than 4 hours	
**Using the computer?**							
**On social networks (e.g. WhatsApp, Instagram, Viber, Messenger)?**							
**Talking on your cell phone?**							
**DURING the quarantine, how many hours per day do you spend:**							
**Watching TV?**	Less than 30 minutes	30 minutes to 1 hour	1 to 2 hours	2 to 3 hours	3 to 4 hours	More than 4 hours	
**Using the computer?**							
**On social networks (e.g. WhatsApp, Instagram, Viber, Messenger)?**							
**Talking on your cell phone?**							

THE MOOD SELF-ASSESSMENT SECTION				
**Before the quarantine, how often did you feel:**				
**Calm?**	Not at all	Somewhat	Moderately	Very much so
**Rested?**				
**Content?**				
**Anxious (tense, preoccupied)?**				
**Angry (irritable)?**				
**Scared?**				
**Discouraged about the future?**				
**Sad?**				
**During the quarantine, how often do you feel:**				
**Calm?**	Not at all	Somewhat	Moderately	Very much so
**Rested?**				
**Content?**				
**Anxious (tense, preoccupied)?**				
**Angry (irritable)?**				
**Scared?**				
**Discouraged about the future?**				
**Sad?**				
